# AI-based denoising improves image quality in HCC volume perfusion CT without affecting Milan classification

**DOI:** 10.1186/s12880-025-02138-6

**Published:** 2026-01-02

**Authors:** Patrick Ghibes, Reza Dehdab, Jan Michael Brendel, Saif Afat, Arne Estler, Christoph Artzner, Konstantin Nikolaou, Andreas Brendlin

**Affiliations:** 1 Department for Diagnostic and Interventional Radiology, Universitätsklinikum Tübingen, Hoppe-Seyler-Strasse 3, 72076 Tübingen, Germany; 2 Department for Radiology, Diakonie Klinikum Stuttgart, Stuttgart, Germany; 3 Cluster of Excellence iFIT (EXC 2180) “Image-Guided and Functionally Instructed Tumor Therapies”, Faculty of Medicine, Eberhard Karls University, Tübingen, Germany

**Keywords:** Volume perfusion computed tomography, Hepatocellular carcinoma, AI-based denoising

## Abstract

**Background:**

Noise and artifacts often compromise image quality and diagnostic accuracy in liver Volume Perfusion CT (VPCT), which can impact clinical decision-making in the diagnosis of hepatocellular carcinoma (HCC). AI-based denoising can potentially improve image quality in computed tomography imaging. Therefore, we aimed to evaluate the effects of AI denoising (AID) on image quality and Milan classification in VPCT compared to standard methods.

**Methods:**

VPCT examinations from 100 patients acquired between 2017 and 2021 were retrospectively included in the analysis. Perfusion maps were reconstructed using original data (Origin), vendor-specific median filtration (Vendor), and AID. Two radiologists independently scored subjective image quality (image quality, diagnostic confidence, contrast, and sharpness). Objective image quality parameters (CT numbers, noise, contrast-to-noise ratios) and diameters of all HCC lesions were measured across all datasets. Additionally, the Milan classification was determined for each patient.

**Results:**

AID enhanced subjective image quality (0.48 ± 0.29) compared to Origin (-0.31 ± 0.29, p-value < 0.001) and Vendor (-0.17 ± 0.24, p-value < 0.001). CNR was higher in AID (27.40 ± 2.98) compared to Origin (16.98 ± 1.54, *p* < 0.001) and Vendor (19.43 ± 1.79, *p* < 0.001). No significant differences were found regarding lesion diameter between Origin, Vendor, and AID (*p* > 0.999). The number and localization of HCC lesions were equal between Origin, Vendor, and AID. The different reconstruction methods did not affect Milan classification.

**Conclusions:**

AID enhances the image quality of HCC liver VPCT without compromising diagnostic capabilities and may support the evaluation of VPCT in the diagnosis of HCC.

**Supplementary Information:**

The online version contains supplementary material available at 10.1186/s12880-025-02138-6.

## Introduction

Hepatocellular carcinoma (HCC) is a malignant tumor originating from the liver and has become an increasingly global health concern due to its rising incidence and high mortality rate [1, 2]. Accurate diagnosis of HCC lesions is vital for treatment decisions. Magnetic resonance imaging (MRI) or multiphase contrast-enhanced computed tomography (CT) is a commonly used imaging technique for HCC diagnosis and staging [3, 4]. Volume perfusion computed tomography (VPCT) has become increasingly important in oncological liver imaging, particularly when the characteristic early enhancement of HCC lesions in the arterial phase of MRI or CT is not detectable [5, 6].

A key disadvantage of VPCT is the high radiation exposure compared to multiphasic CT or radiation-free MRI [7]. However, the diagnostic accuracy of VPCT relies on the quality of acquired CT images, with noise being a key factor that limits both image quality and diagnostic confidence. Therefore, a compromise must always be found between the necessary radiation dose and image quality [8]. Furthermore, image artifacts such as noise may accumulate in the process of 4-dimensional VPCT coregistration, which may further hinder the diagnostic accuracy of the method even in the context of modern iterative reconstruction. To tackle this issue, noise reduction through postprocessing methods can help to improve the image quality [9]. In VPCT, median filtration of coregistered iterative reconstructed 4D datasets is the traditional method of noise reduction; however, low-contrast lesion detection may be problematic, especially concerning small hepatic or pancreatic masses [10, 11].

In standard CT imaging, AI-based Denoising (AID) algorithms have gained attention lately for improving image quality by reducing noise while preserving anatomical details [12–14]. However, novel AI image quality improvement algorithms also bring new challenges, such as potential spatial resolution deterioration and blurring, which may negatively impact reporting [15]. Therefore, prominent review articles advocate for rigorous use-case-level validations of these solutions [16]. Only a few studies have investigated the use of AI denoising in abdominal imaging issues. All of them have demonstrated a clear improvement in image quality [17–19]. Inoue et al. examined the application of AI denoising in multiphase liver CT. In their study, comparable image quality was achieved despite a 50% reduction in radiation dose [20]. Despite encouraging reports that deep-learning denoising can maintain or approximate standard-dose/contrast CT perfusion image quality in both preclinical and clinical settings, and systematic reviews suggesting that AI may reduce iodinated contrast requirements, no study has validated its impact on image quality and Milan classification in liver VPCT [21–23]. Addressing this gap is clinically crucial. A curative treatment option for patients with HCC is liver transplantation. However, this treatment option shows good long-term recurrence-free survival only when the patients have a single tumor lesion smaller than 5 cm in diameter, or a maximum of three tumor lesions, each with a diameter of up to 3 cm [24]. Incorrectly detected lesions in VPCT or inaccurate size measurements can prevent a curative treatment in patients with HCC.

Therefore, we wanted to evaluate the performance of an AID algorithm in the context of iteratively reconstructed 4D-coregisterred liver VPCT against further unprocessed data and standard, vendor-specific median-filtration. We hypothesized that AID can significantly improve image quality without affecting critical diagnostic parameters such as lesion size or Milan criteria.

## Materials and methods

### Ethical consideration

This monocentric retrospective study was approved by the institutional review board (study #167/2022BO2) with a waiver for the need for informed consent and was conducted in accordance with the ethical standards outlined in the Declaration of Helsinki, as revised in 2013.

### Study design and patient population

**Fig. 1:**
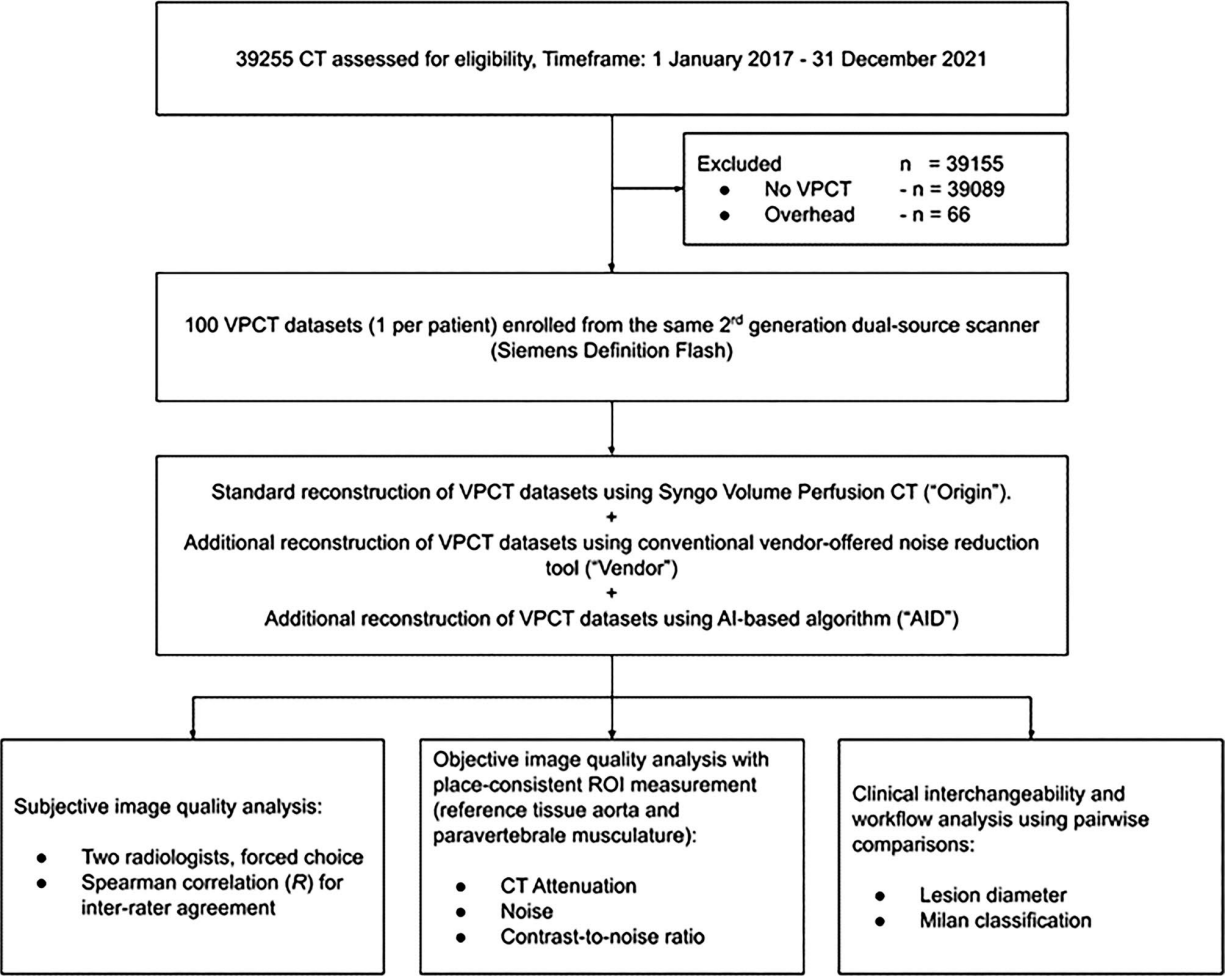
Study Flowchart

An a priori power analysis using the software solution G*Power (ver. 3.1.9.7, Franz Faul, University of Kiel, Germany) determined the necessary total sample size for this experiment (f = 0.162, α = 0.05, 1-β = 0.95) to be 100 patients [25]. The calculated case number also guarantees that patients with liver cirrhosis, with primary suspicion of HCC, or patients for therapy monitoring are included in the patient population. For this purpose, all patients from our Radiology department were screened for eligibility from 01 January 2017 to 01 January 2022. We excluded all examinations other than VPCT. The first 100 VPCT examinations were included in chronological order. Additionally, per patient, only the initial VPCT was included. From the 100 patients thus included, we collected demographic data from the clinical information system, as well as their CT dose index (CTDIvol in mGy) and dose-length products (DLP in mGy*cm). See Fig. 1 for a detailed explanation of the study workflow, the inclusion process, and the conducted experiments.

### Image acquisition and reconstruction parameters

All VPCT examinations were performed on a second-generation dual-energy CT scanner (SOMATOM Definition Flash; Siemens Healthineers, Erlangen, Germany). The CT protocol consisted of a non-enhanced abdominal low-dose CT (40 mAs, 100 kV, slice thickness = 5.0 mm, collimation 128 m × 0.6 mm, tube rotation time 0.5 s, pitch 0.6) for planning the subsequent VPCT. Depending on the liver size, the VPCT was planned with a field of view (FOV) of 150–180 mm to cover the entire liver. VPCT parameters were 100 mAs, 80 kV, 64 mm × 0.6 mm collimation, and 26 CT whole-coverage scans of the entire liver parenchyma. Acquisition time was 40s. The patients were advised to breathe shallowly during the acquisition. 50 mL of contrast medium (Ultravist 370, Bayer Vital, Leverkusen, Germany) was injected at a flow rate of 4.5 mL/s, followed by a saline flush of 50 mL of NaCl with a flow rate of 4.5 mL/s and a start delay of 7 s. The contrast medium was injected through an antecubital vein cannula using a dual-head power injector (CT Stellant, Medrad, Indianola, PA, USA). For postprocessing and perfusion analysis of VPCT, a set of axial images with a slice thickness of 3 mm was reconstructed without overlap using a smooth tissue convolution kernel (B10f). All image data sets were transferred to a dedicated workstation (Syngo MMWP, VE 36 A, Siemens Healthineers, Erlangen, Germany). As the second step, perfusion was evaluated with commercial software (Syngo Volume Perfusion CT (Siemens Healthineers, Erlangen, Germany). Three perfusion evaluations were performed for each data set. The first evaluation did not include noise reduction (“Origin”) of the source data. For the second evaluation (“Vendor”), a conventional noise reduction method offered by the vendor (Siemens Healthineers, Erlangen, Germany) was applied to the source data. For the third evaluation, all source data images were processed using a novel, AI-based denoising (“AID”) algorithm (ClariCT.AI; version 1.2.1, ClariPi Inc., Seoul, South Korea, 2023), which is based on a modified U-net-type convolutional neural network model (CNN). We used neutral blending (edge/noise 0), which balances edge preservation and noise suppression without aggressive smoothing or edge sharpening, per default vendor guidance. Automated motion correction was applied for all evaluations. According to the vendor’s specifications, arterial liver perfusion (ALP) and portal venous perfusion (PVP) are calculated using the time of peak splenic enhancement as the separation point between the arterial and portal-venous phases. Therefore, regions of interest (ROI) were drawn in the portal vein and the splenic parenchyma. ROI size and ROI position were the same for all three evaluations.

### Subjective image quality comparison

The patient datasets were anonymized and randomized by a group member who was not otherwise associated with diagnostic confidence analysis. Two radiologists with 3 and 5 years of experience in liver VPCT analysis, respectively, independently evaluated four image quality parameters: overall image quality, diagnostic confidence, sharpness, and contrast. Evaluations were conducted in a blinded, randomized, forced-choice format using the open-source software ViewDex (ver. 3.0, Angelica Svalkvist, Sune Svensson, Tommy Hagberg, and Magnus Båth, Sahlgrenska University Hospital, Gothenburg, Sweden [26]). For each assessment, paired datasets were displayed side-by-side, with the reference image on the left and the image to be rated on the right. Ratings were assigned as − 1 (inferior), 0 (equal), or + 1 (superior) based on the subjective comparison [27]. All image sets were initially presented using a standard soft tissue window (center: 50 HU; width: 350 HU), with the option for individual windowing adjustments during review. The scores from all evaluations were averaged per dataset to generate a comparable semiquantitative score.

### Objective image quality comparison

For objective image quality assessment, the VPCT data sets per patient (Origin, Vendor, and AID) were imported into the open-source software ImageJ distribution FIJI (Version 1.53 k, Wayne Rasband, National Institutes of Health-NIH, Maryland, USA). Two regions of interest (ROI) were manually positioned in the paravertebral musculature as a reference tissue to the liver parenchyma and the abdominal aorta at the anatomical level of the portal vein bifurcation for the Origin image data set. Mean CT numbers were calculated in Hounsfield units (HU) and the standard deviation (SD). Subsequently, the selected ROIs were automatically transferred to the Vendor and AID image data. The pooled SD of the mean CT numbers of the paravertebral muscle and the abdominal aorta was defined as a quantitative parameter for noise and was calculated using the following formula.

$$\:{SD}_{pooled}=\:\sqrt{\frac{\sum\:{SSD}_{i}}{\sum\:{r}_{i}-1}}$$

SSD_i_ is the sum of the sqares of the standard deviation. r_i_ is the number of the repeated measurments on the sample.

Contrast-to-noise Ratio (CNR) was calculated using the following formula:

$$\:\boldsymbol{C}\boldsymbol{N}\boldsymbol{R}=\:\frac{\mathrm{M}\mathrm{e}\mathrm{a}\mathrm{n}\:\mathrm{A}\mathrm{o}\mathrm{r}\mathrm{t}\mathrm{a}-\mathrm{M}\mathrm{e}\mathrm{a}\mathrm{n}\:\mathrm{p}\mathrm{a}\mathrm{r}\mathrm{a}\mathrm{v}\mathrm{e}\mathrm{r}\mathrm{t}\mathrm{e}\mathrm{b}\mathrm{r}\mathrm{a}\mathrm{l}\:\mathrm{m}\mathrm{u}\mathrm{s}\mathrm{c}\mathrm{l}\mathrm{e}\:\left(\mathrm{H}\mathrm{U}\right)}{\mathrm{P}\mathrm{o}\mathrm{o}\mathrm{l}\mathrm{e}\mathrm{d}\:\mathrm{A}\mathrm{o}\mathrm{r}\mathrm{t}\mathrm{a}\:\&\:\mathrm{p}\mathrm{a}\mathrm{r}\mathrm{a}\mathrm{v}\mathrm{e}\mathrm{r}\mathrm{t}\mathrm{e}\mathrm{b}\mathrm{r}\mathrm{a}\mathrm{l}\:\mathrm{m}\mathrm{u}\mathrm{s}\mathrm{c}\mathrm{l}\mathrm{e}\:\left(\mathrm{S}\mathrm{D}\right)}$$

### Clinical interchangeability

The lesion size and the number of HCC lesions were determined as further quantitative parameters by the same two radiologists with 3 and 5 year experience in VPCT analysis, as the size and number of lesions are crucial for the Milan classification. For this purpose, all HCC or HCC-suspected lesions (lesions with arterial phase hyperenhancement and non-peripheral washout) were identified in the original findings. In a second step, the lesions were localized and measured on the same slice position for all three VPCT data sets (Origin, Vendor, AID) and assigned to the corresponding liver segment. Findings that could not be localized retrospectively in the initial image data were excluded from further evaluation. The Milan classification was determined based on the diameters measured for the three reconstruction methods. The lesions were measured in the arterial liver perfusion map. For this purpose, the size of the hyperenhanced area was determined, which which marked in red by the VPCT analysis software.

### Statistical analysis

Statistical computations and graphics were generated in GraphPad Prism 9.3.1 for Windows (GraphPad Software, San Diego, CA, USA). We first assessed whether each variable followed a normal distribution using the Shapiro–Wilk test. Variables with normal distributions are reported as mean ± SD, whereas those that were not normally distributed are presented as median (interquartile range). For inferential analyses, we applied a mixed-effects model, which allows to detect fixed and random effects of the patient collective, incorporating the Greenhouse–Geisser correction whenever the assumption of sphericity was breached. To control the false discovery rate in our post hoc multiple comparisons, we employed the two-stage step-up procedure developed by Benjamini, Krieger, and Yekutieli to identify as many significant comparisons as possible while still maintaining a low false positive rate. Statistical significance was defined as an adjusted p-value of 0.05 or lower. Finally, inter-rater reliability for the subjective image–quality scores was quantified using Spearman’s rho (ρ), with ρ values indicating the following levels of agreement: 0–0.20, negligible; 0.21–0.40, weak; 0.41–0.60, moderate; 0.61–0.80, strong; and 0.81–1.00, very strong.

Additionally, statistical Analysis Inter- and intra-rater reliability regarding subjective image-qualitiy scores were assessed using the Prevalence-Adjusted Bias-Adjusted Kappa (PABAK) to account for prevalence imbalances in the ordinal ratings. To obtain robust reliability estimates for granular subgroups (Item×Comparison, *N* ≈ 100) while avoiding overfitting to small sample sizes, we implemented a hierarchical Bayesian Beta-Binomial model. The model utilized partial pooling to shrink estimates of smaller subgroups toward the group mean, reducing the impact of statistical noise. Crucially, to prevent high intra-rater consistency from inflating inter-rater estimates, we employed a stratified hierarchy: separate hyperpriors (ϕ,κ) were modeled for inter-rater and intra-rater comparisons. Global and item-level estimates (large N) were calculated analytically using independent Beta posteriors. Subgroup estimates were computed using Markov Chain Monte Carlo (MCMC) sampling with the No-U-Turn Sampler (NUTS) via the PyMC library (v5.x). We report the posterior mean and the conservative lower bound of the 95% Highest Density Interval (HDI) to ensure strict reporting of reliability.

## Results

### Study population

This study initially included one hundred patients (see Fig. 1). The VPCT image data could be evaluated in all patients. The mean age of the patients was 62 ± 12 years. The mean CTDIvol was 58 ± 2 mGy, and the mean overall DLP was 1062 ± 95 mGy*cm (see Table 1).

**Table 1:** Baseline characteristics of the investigated patient population

Characteristics	Overall	Female	Male
Age (years)	62 ± 12	63 ± 7	62 ± 13
Body Diameter (cm)	30 ± 3	27 ± 3	31 ± 2
Contrast agent (ml)	102 ± 33	98 ± 34	102 ± 33
mAs reference	100	100	100
CDTI (mGy)	58 ± 2	59 ± 2	58 ± 2
DLP (mGy*cm)	1062 ± 95	1047 ± 222	1064 ± 62
SSDE (mGy)	1286 ± 160	1449 ± 238	1264 ± 133

HU = Hounsfiled Unit; SD = standard deviation; CNR = Contrast-to-noise ratio; AID = AI Denoising; n/a = not applicable

### Subjective image quality comparison

**Fig. 2:**
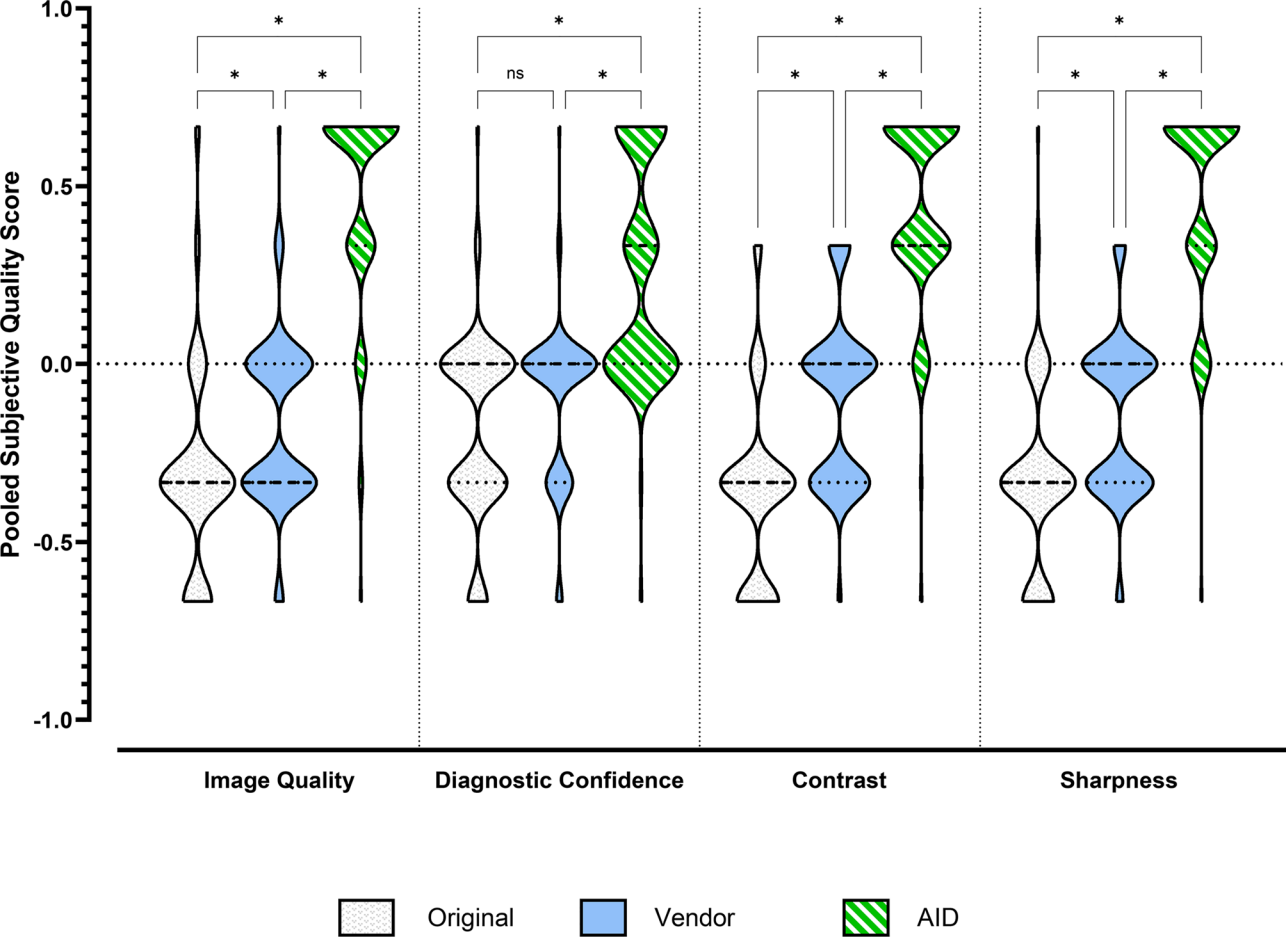
Rater-specific subjective image quality ratings and reliability/agreement metrics for Original (white bar), Vendor (blue bar), and AID (green bar). Significant differences are marked with an asterisk

Tables S1, Table S2, Table S3 and Fig. 2 summarize the results of the subjective evaluation of the image quality, diagnostic confidence, sharpness of the lesions, and contrast. We measured very strong inter-rater agreement in the subjective image quality comparisons (Rho = 0.84, 95% CI (0.80 to 0.87), each *p* < 0.001). Inter-rater reliability was consistently high across reconstruction methods and image quality criteria. At the global level, the Bayesian prevalence-adjusted bias-adjusted kappa (PABAK) for the two raters was 0.95, with a 95% highest density interval (HDI) of 0.93–0.97. On the item level, inter-rater PABAK means ranged from 0.93 to 0.97 (Contrast: 0.95; Diagnostic Confidence: 0.97; Image Quality: 0.93; Sharpness: 0.93), and the corresponding 95% HDI lower bounds ranged from 0.89 (Image Quality, Sharpness) to 0.93 (Diagnostic Confidence). Across all granular comparisons, the lowest conservative estimate occurred for Image Quality in the Vendor vs. AID comparison (Rater 1 vs. Rater 2), with a PABAK mean of 0.89 and a 95% HDI of 0.80–0.96. Importantly, even this most challenging comparison showed a lower 95% HDI bound of 0.80, indicating at least strong agreement under conservative Bayesian interpretation. Across all other inter-rater comparisons, 95% HDI lower bounds were ≥ 0.82, and PABAK means ranged from 0.90 to 0.99. Intra-rater reliability was near-perfect throughout, with intra-rater PABAK means ≥ 0.99 and 95% HDI lower bounds ≥ 0.97 across all reconstruction methods and image quality criteria.

In the pooled data, Mixed effects analysis revealed significant interactions between Image Quality, Diagnostic Confidence, Contrast, and Sharpness for Origin, Vendor, and AID (F [4.89, 528] = 161; η² = 0.444). The data are normally distributed, so all data are presented as means with standard deviations in the following. In the post-hoc tests, the parameter image quality for AID was rated significantly higher compared to Origin (0.48 ± 0.26 vs. -0.31 ± 0.29, *p* < 0.001) as well as to dataset Vendor (0.48 ± 0.29 vs. -0.17 ± 0.24, *p* < 0.001). There were also significant differences between the dataset Vendor and Origin (-0.17 ± 0.24 vs. -0.31 ± 0.29, *p* = 0.023). The diagnostic confidence of the AID dataset was rated significantly better than that of the Vendor or Origin dataset; for example, AID 0.27 ± 0.31 vs. Origin − 0.19 ± 0.26, *p* < 0.001. No significant differences were detected between the Vendor and Origin ratings. Similar results were found for sharpness and contrast. AID showed significantly higher ratings than Vendor and Origin. Sharpness was rated 0.47 ± 0.27 for AID vs. -0.14 ± 0.23 for Vendor, *p* < 0.001. Significant differences were found between Vendor and Origin for sharpness and contrast; for example, Vendor: -0.10 ± 0.24 vs. Origin: -0.33 ± 0.26, *p* < 0.001 for the parameter contrast.

### Objective image quality comparison

**Fig. 3:**
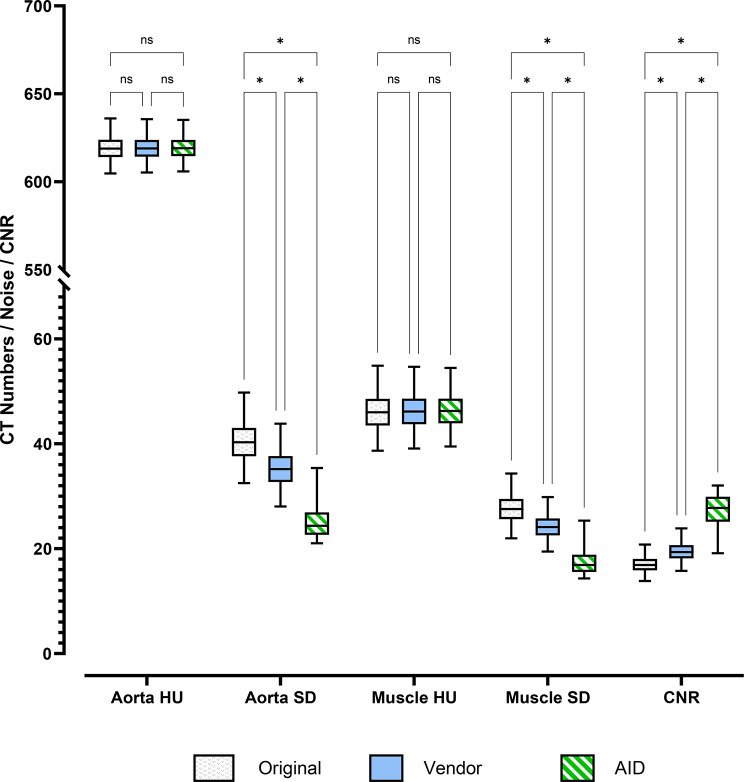
Data distribution and pairwise comparisons for Original (white bar), Vendor (blue bar), and AID (green bar) of objective image quality metrics of reference tissue (Aorta, paraspinal muscles): CT numbers (HU), Noise (SD), and Contrast-to-Noise Ratios (CNR). ns = not significant, * = significant

Mixed effects analysis revealed significant interactions between CT numbers, SD, and CNR for Origin, Vendor, and AID (F [1.02, 101] = 21051; η² = 0.127). The data are normally distributed, so all data are presented as means with standard deviations in the following.Within the CT numbers, post-hoc analysis revealed no significant differences between Origin, Vendor, and AID image data in the paravertebral musculature and the abdominal aorta (see Table S4 and Fig. 3). For example, the mean HU in the abdominal aorta for Origin was 619.19 ± 6.95 and 619.39 ± 6.50 for AID (*p* = 0.253). Significant differences were found for noise between Origin, Vendor, and AID. Noise in the abdominal aorta was 40.44 ± 3.83 for Origin and 35.29 ± 3.51 for Vendor (*p* < 0.001), which was significantly lower for AID (25.01 ± 2.95, *p* < 0.001). We measured significantly higher CNR for AID (27.40 ± 2.98), which was approximately 50% higher than Vendor (19.43 ± 1.79, *p* < 0.001) and approximately twice as high as Origin (16.98 ± 1.54, *p* < 0.001).

### Clinical interchangeability

**Fig. 4:**
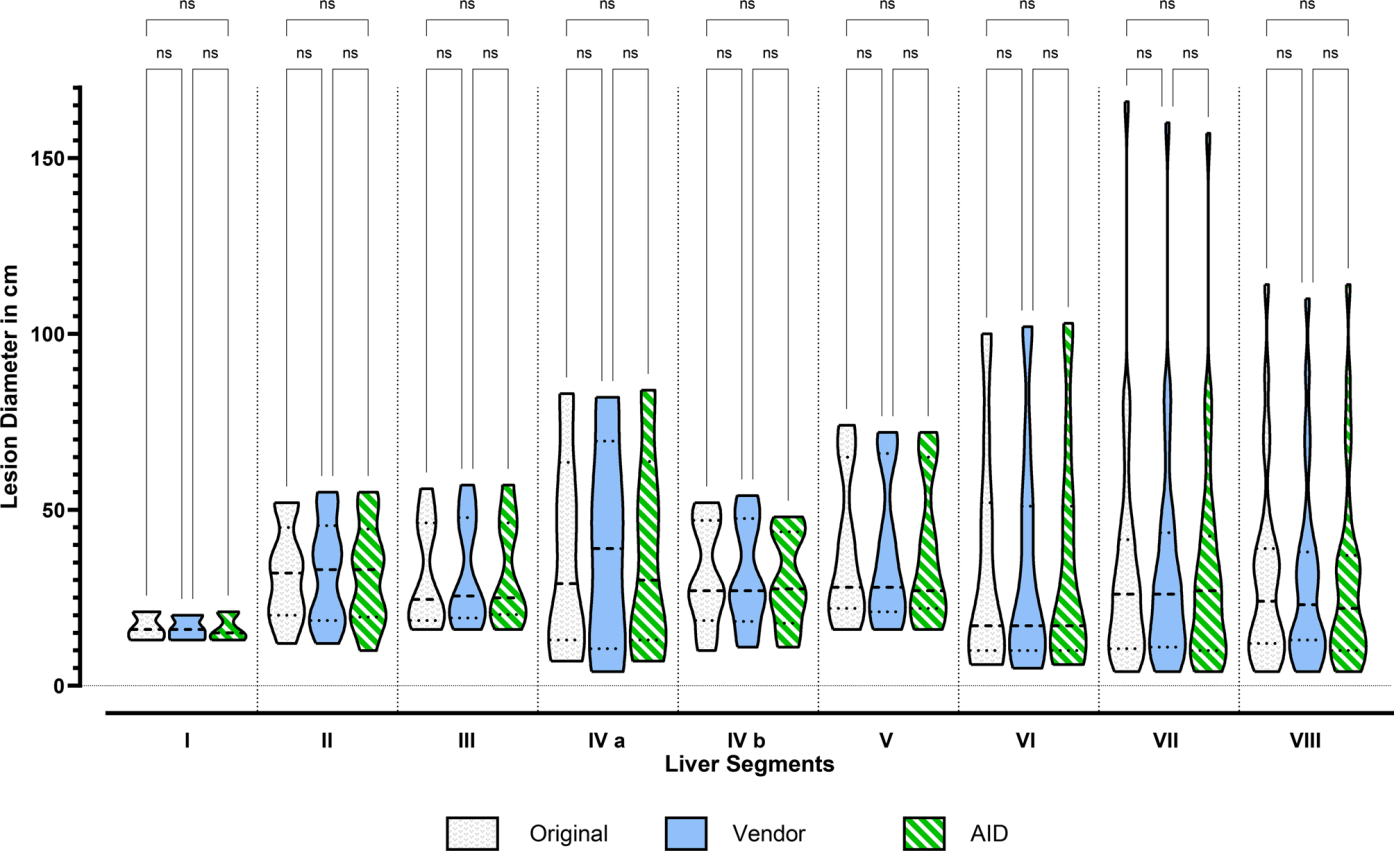
Data distribution and pairwise comparisons of clinical interchangeability metrics: Lesion diameters per segment and series. ns = not significant, * = significant

Seventy patients were inside Milan, and 30 were outside Milan. A total of 116 liver lesions were found and evaluated for all three reconstructions (Origin, Vendor, and AID). Most liver lesions were located in segment VII (*n* = 29). Only three liver lesions were found in liver segment I. The three reconstructions properly imaged all present lesions. Furthermore, no other liver parenchymal lesions were detected in the Vendor or AID images. Table S5 and Fig. 4 summarize the results of the standardized evaluation of liver lesions by liver segment. No significant difference was found between the three reconstruction methods. The largest deviation was observed in segment IVa, where we measured average lesion diameters of 37 mm ± 29 mm for Origin, 40 mm ± 31 mm for Vendor, and 37 mm ± 30 mm for AID (p-value > 0.999).

**Fig. 5:**
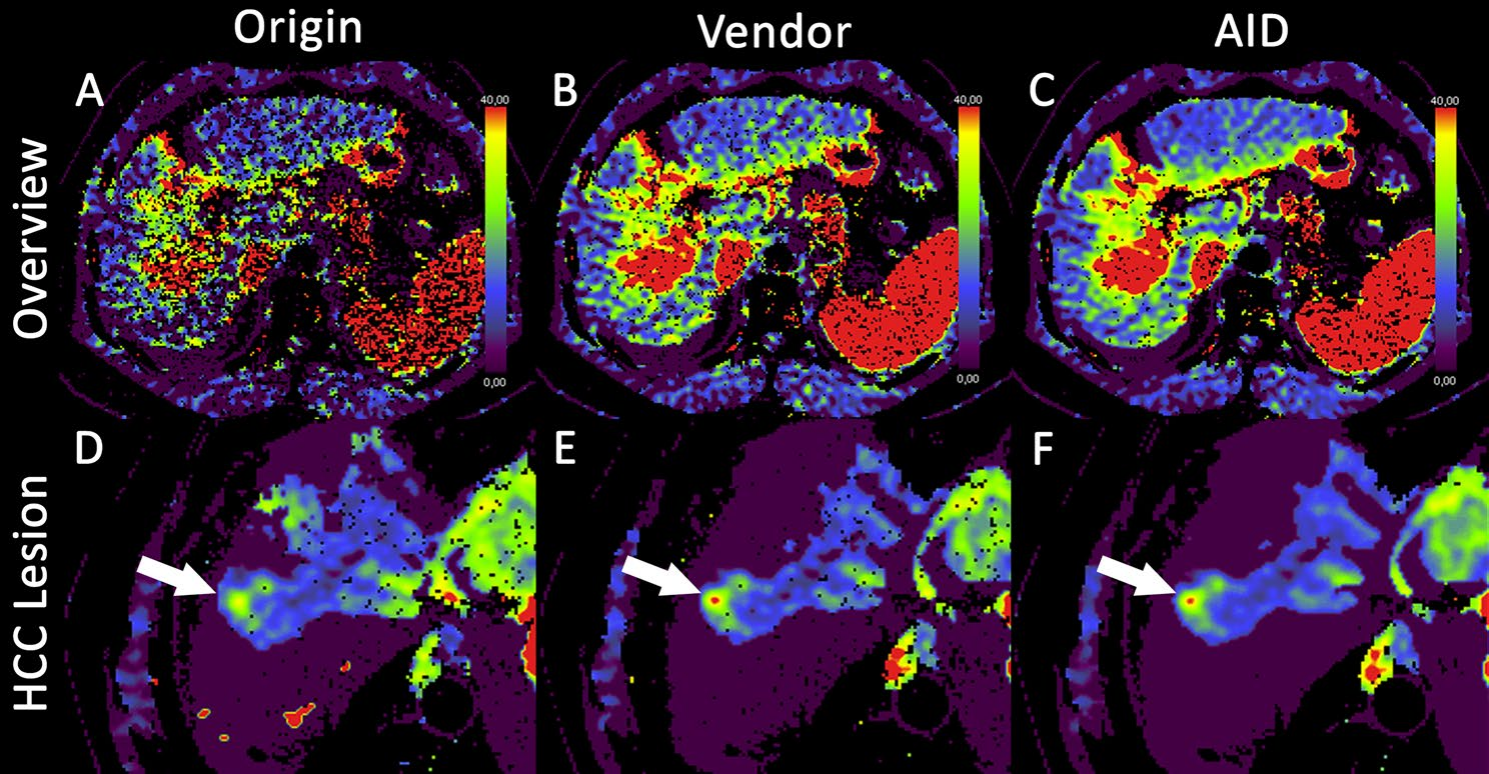
Differences in imaging quality for the arterial liver perfusion map for Origin (**A**), Vendor (**B**), and AID (**C**) image reconstruction. Pathohistologically confirmed HCC lesion (white arrows) of the same patient in liver segment VII for Origin (**D**), Vendor (**E**), and AID (**F**) image reconstruction

**Fig. 6:**
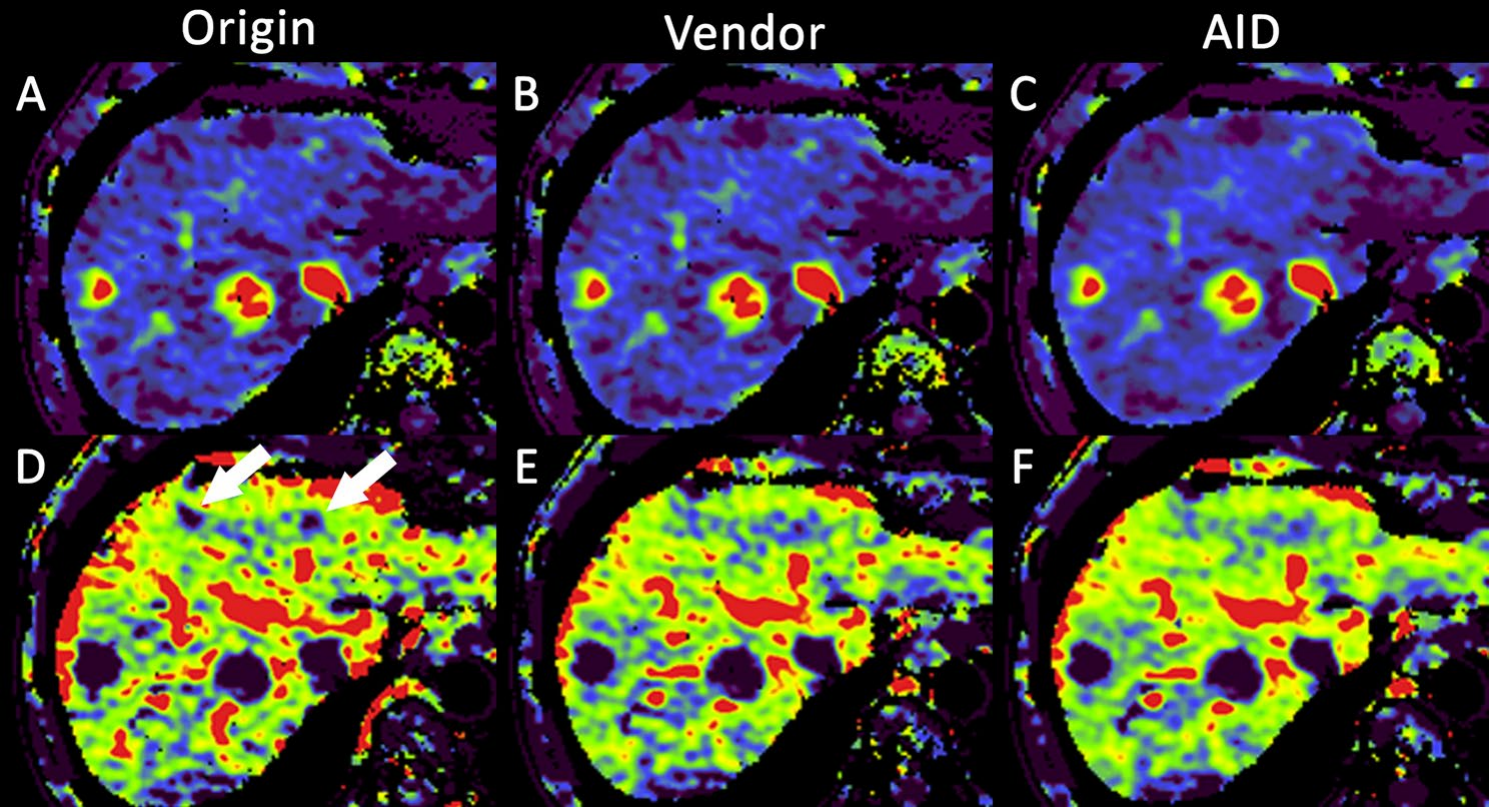
67-year-old patient with two HCC lesions in liver segment VII, which are visible in the arterial liver perfusion map for Origin (**A**), Vendor (**B**), and AID (**C**) image reconstruction. Portal vein perfusion maps show two suspicious lesions with washout in liver segment IVa for Origin (**D**, white arrows) image reconstruction. The suspicious lesions are not visible in the Vendor (**F**) and AID (**G**) image reconstructions, indicating a potential for false positives in Origin

The Milan criteria were evaluated, and the three reconstruction methods showed no significant differences. Figures 5 and 6 illustrate the differences in image quality, with fewer black artifacts and less noise, for AID compared to Vendor and Origin.

## Discussion

Hepatocellular carcinoma (HCC) remains a significant global health burden, where accurate imaging is essential for diagnosis and treatment planning. Volume perfusion CT (VPCT) offers functional insight but is limited by high radiation exposure and image noise. In this context, AI-based denoising may offer a promising solution, yet its impact on HCC-specific VPCT workflows has not been previously explored. Therefore, we aimed to evaluate the performance of an AI denoising (AID) algorithm on VPCT and its possible influence on Milan classification in the context of HCC. We hypothesized that AID enhances the image quality of standard VPCT without compromising diagnostic accuracy. Our findings demonstrate that using deep learning noise reduction methods improves image quality, diagnostic confidence, sharpness, and contrast compared to conventional noise reduction methods and imaging analysis without noise reduction. Additionally, AID did not impact the Milan classification.

Several studies have demonstrated that AID can enhance imaging quality and reduce noise in various imaging applications. Hong et al. applied AID to coronary angiography CT and demonstrated that noise was significantly reduced. In addition, a significant improvement in SNR and CNR was achieved [28]. Our results also demonstrate a significant improvement in CNR, possible to observe due to a lower standard deviation of the signal in the aorta., which was approximately 50% higher for AID compared to Vendor. Similar results were shown by Lee et al. and Nam et al. for ultra-low-dose chest CT and pediatric abdominal CT [29, 30]. In our study, AID significantly reduced image noise and artifact burden, thereby improving image quality compared to Origin and Vendor. These findings align with previous publications that have investigated AI noise reduction capabilities in various patient cohorts [14, 31]. However, only a few studies have investigated AID in liver imaging, and to our knowledge, we are the first to investigate AID in the context of VPCT [32, 33]. Despite technical advances in CT scanner technology and postprocessing software, VPCT of the liver causes significantly higher radiation exposure in the target volume than conventional multiphasic abdominal CT. This is primarily due to the high temporal and spatial resolution required [7, 34]. Standardized imaging protocols and reference values are often lacking due to limited study availability. The results of these studies are usually specific to their imaging protocols. Individual studies have investigated low-dose acquisition protocols; however, these protocols have shown limitations for clinical use due to their negative impact on various perfusion parameters [7, 35]. Bevilacqua et al. demonstrated that radiation exposure could be reduced at scan delays greater than 10s, but portal venous blood flow perfusion maps could no longer be evaluated due to artifact burden [8]. The objective image quality analysis revealed that the CT numbers (HU) were not affected by Vendor or AID. This result is important because a change in the CT numbers could affect the evaluation of the VPCT; in the worst case, HCC suspicious lesions in the liver parenchyma could no longer be detected. AID shows a significantly higher CNR compared to Vendor and Origin. A significant noise reduction was also demonstrated in various studies investigating deep-learning reconstructions in other anatomical regions; for example, Hong et al. demonstrated that deep-learning algorithms can increase CNR significantly in coronary CT angiography [28].

The evaluation of the individual HCC lesions showed that the diameter of the individual lesions was not significantly changed by postprocessing using the Vendor and AID algorithms. This is an important fact, because VPCT is primarly used for the detection of HCC lesion. So the purpose of the denoising algorithm is not to alter signal values, as this could have directly affect of perfusion maps and the size of HCC lesion.The results ouf our study confirms, that the Milan criteria, which have a decisive influence on the therapy of HCC, did not change in any of the patients. It is important to note, that it the denoising algortihm can still cause false positives or false negatives findings. Therefore, every questionable finding should be critically evaluated, and all available contrast phases should be reviewed. In cases of doubt about the presence of an HCC lesion, it may be advisable to review the images without denoising. If there are still concerns, additional modalities such as MRI or contrast-enhanced ultrasound should be considered. Imaging of HCC remains challenging because the sensitivity and accuracy of an imaging method depend not only on the size of the lesion but also on the histopathology and the scoring system used. Studies have shown that MRI with liver-specific protocols is more sensitive than single-phase contrast-enhanced CT. Lee et al. demonstrated that a sensitivity of up to 92% can be achieved by MRI in the detection of HCC lesions when gadolinium is used [36]. According to Rostambeigi et al., if only a contrast-enhanced CT is performed to diagnose HCC, up to one-fifth of lesions will be missed, resulting in incorrect classification into the Milan criteria in up to 14% of patients [37]. VPCT has similar sensitivity compared to MRI in detecting HCC lesions [38]. Kurucay et al. compared the diagnostic performance of VPCT and MRI in detecting HCC lesions. They found that VPCT was able to detect all lesions, as all were identified due to high-grade arterial blood supply. In contrast, MRI required the combination of all acquired sequences, as not all lesions were detectable in the arterial phase [39]. Fischer et al. examined 26 patients with liver cirrhosis using VPCT to detect the presence of HCC. The study achieved a sensitivity and specificity of nearly 100% for the detection of HCC [40]. Despite advances in MRI imaging, VPCT is particularly valuable in HCC imaging for patients unsuitable for MRI examination [41]. The observation that AI-based denoising enhances image quality without altering lesion size or the Milan criteria suggests its potential application in optimizing VPCT protocols to reduce radiation exposure in a second step [22]. Several studies have demonstrated that AI denoising can substantially decrease radiation dose while maintaining diagnostic image quality [9, 42, 43]. Therefore, further research is warranted to validate these findings and establish standardized protocols.

This study has several limitations. First, this was a retrospective study with 100 patients. A major limitation of retrospective study design is that a selection bias cannot be excluded. Further prospective studies are necessary to confirm the impact of AI denoising on VPCTs. Second, the VPCT evaluations depend highly on vessel and liver parenchyma definition ROIs, which must be done manually. Despite the uniform size and positioning on the same image slice, discrepancies due to the possible influence of postprocessing on the results cannot be excluded. However, it is encouraging that we did not find significant differences between lesion diameters. Thirdly, the imaging parameters pertain to one high-end dual-energy CT scanner with a specific imaging protocol. Using different machines or adjusting imaging parameters is expected to impact image quality significantly. Therefore, the results of this study may be specific to our setup, and their generalizability may be limited. Further studies with a comparison of different CT scanners may be necessary. Fourth, this study investigated a specific AID algorithm. The applicability of our results to other AID algorithms may be limited, as algorithm performance can vary, which may impact lesion size measurements and Milan classification. Fifth, We only investigated HCC lesions in this study. The detection of other lesions (such as hemangiomas or metastases) and their differentiation were not part of this study and should be the focus of future research. Finally, we did not include an external validation cohort, which further limits the generalizability of our findings and should be addressed in future multicenter studies.

## Conclusions

AI-based denoising improves image quality in VPCT for HCC without altering lesion size or Milan classification. This technology may enhance diagnostic confidence and treatment planning while supporting dose-optimization strategies. With further multicenter or multi-scanner validation, AI denoising should be considered for integration into standard VPCT workflows.

## Supplementary Information

Below is the link to the electronic supplementary material.

Supplementary Material 1

## Data Availability

The datasets used and/or analysed during the current study are available from the corresponding author on reasonable request.

## Abbrevations

AI: Artificial intelligence
AID: AI-based Denoising Algorithm
ALP: Arterial liver perfusion
CTDI_vol_: CT Dose Index
CNR: Contrast-to-Noise Ratio
CT: Computed Tomography
DLP: Dose-Length Product
HCC: Hepatocellular carcinoma
HU: Hounsfield Unit
MRI: Magnetic Resonance Imaging
PVP: Portal venous perfusion
ROI: Region of Interest
SD: Standard deviation
SNR: Signal-to-Noise Ratio
VPCT: Volume perfusion computed tomography

## References

[1] Orci LA, Sanduzzi-Zamparelli M, Caballol B, Sapena V, Colucci N, Torres F, et al. Incidence of hepatocellular carcinoma in patients with nonalcoholic fatty liver disease: A systematic Review, Meta-analysis, and Meta-regression. Clin Gastroenterol Hepatology: Official Clin Pract J Am Gastroenterological Association. 2022;20(2):283–e9210. 10.1016/j.cgh.2021.05.002 33965578

[2] Forner A, Reig M, Bruix J. Hepatocellular carcinoma. Lancet (London England). 2018;391(10127):1301–14. 29307467 10.1016/S0140-6736(18)30010-2

[3] Ayuso C, Rimola J, Vilana R, Burrel M, Darnell A, García-Criado Á, et al. Diagnosis and staging of hepatocellular carcinoma (HCC): current guidelines. Eur J Radiol. 2018;101:72–81. 29571804 10.1016/j.ejrad.2018.01.025

[4] Marrero JA, Kulik LM, Sirlin CB, Zhu AX, Finn RS, Abecassis MM, et al. Diagnosis, Staging, and management of hepatocellular carcinoma: 2018 practice guidance by the American association for the study of liver diseases. Hepatology (Baltimore MD). 2018;68(2):723–50. 29624699 10.1002/hep.29913

[5] Petralia G, Bonello L, Viotti S, Preda L, D’Andrea G, Bellomi M. CT perfusion in oncology: how to do it. Cancer Imaging: Official Publication Int Cancer Imaging Soc. 2010;10(1):8–19. 10.1102/1470-7330.2010.0001 PMC2842179 20159664

[6] Spira D, Schulze M, Sauter A, Brodoefel H, Brechtel K, Claussen C, et al. Volume perfusion-CT of the liver: insights and applications. Eur J Radiol. 2012;81(7):1471–8. 21543180 10.1016/j.ejrad.2011.04.010

[7] Kalarakis G, Perisinakis K, Akoumianakis E, Karageorgiou I, Hatzidakis A. CT liver perfusion in patients with hepatocellular carcinoma: can we modify acquisition protocol to reduce patient exposure? Eur Radiol. 2021;31(3):1410–9. 32876834 10.1007/s00330-020-07206-9

[8] Bevilacqua A, Malavasi S, Vilgrain V. Liver CT perfusion: which is the relevant delay that reduces radiation dose and maintains diagnostic accuracy? Eur Radiol. 2019;29(12):6550–8. 31115620 10.1007/s00330-019-06259-9

[9] Greffier J, Hamard A, Pereira F, Barrau C, Pasquier H, Beregi JP, et al. Image quality and dose reduction opportunity of deep learning image reconstruction algorithm for CT: a Phantom study. Eur Radiol. 2020;30(7):3951–9. 32100091 10.1007/s00330-020-06724-w

[10] Mileto A, Guimaraes LS, McCollough CH, Fletcher JG, Yu L. State of the Art in abdominal CT: the limits of iterative reconstruction algorithms. Radiology. 2019;293(3):491–503. 31660806 10.1148/radiol.2019191422

[11] Laurent G, Villani N, Hossu G, Rauch A, Noel A, Blum A, et al. Full model-based iterative reconstruction (MBIR) in abdominal CT increases objective image quality, but decreases subjective acceptance. Eur Radiol. 2019;29(8):4016–25. 30701327 10.1007/s00330-018-5988-8

[12] Sandfort V, Willemink MJ, Codari M, Mastrodicasa D, Fleischmann D. Denoising multiphase functional cardiac CT angiography using deep learning and synthetic data. Radiol Artif Intell. 2024;6(2):e230153. 38416035 10.1148/ryai.230153 PMC10982910

[13] Nagata M, Ichikawa Y, Domae K, Yoshikawa K, Kanii Y, Yamazaki A, et al. Application of deep Learning-Based denoising technique for radiation dose reduction in dynamic abdominal CT: comparison with Standard-Dose CT using hybrid iterative reconstruction method. J Digit Imaging. 2023;36(4):1578–87. 36944812 10.1007/s10278-023-00808-x PMC10406991

[14] Park M, Hwang M, Lee JW, Kim KI, Ahn C, Suh YJ, et al. Application of a deep Learning-Based Contrast-Boosting algorithm to Low-Dose computed tomography pulmonary angiography with reduced iodine load. J Comput Assist Tomogr. 2025;49(2):288–96. 39438307 10.1097/RCT.0000000000001665

[15] Diwakar M, Kumar M. A review on CT image noise and its denoising. Biomed Signal Process Control. 2018;42:73–88.

[16] Mohammadinejad P, Mileto A, Yu L, Leng S, Guimaraes LS, Missert AD, et al. CT noise-reduction methods for lower-dose scanning: strengths and weaknesses of iterative reconstruction algorithms and new techniques. Radiographics. 2021;41(5):1493–508. 34469209 10.1148/rg.2021200196

[17] Hou P, Feng X, Chen Y, Wang X, Jiang Y, Liu J, et al. Ultra-low-dose hepatic computed tomography with a novel real-time deep learning-based noise reduction algorithm: a prospective cross-sectional analysis of image quality and lesion detection. Quant Imaging Med Surg. 2025;15(8):7006–18. 40785866 10.21037/qims-2025-365 PMC12332563

[18] Bae JS, Lee JM, Kim SW, Park S, Han S, Yoon JH, et al. Low-contrast-dose liver CT using low monoenergetic images with deep learning-based denoising for assessing hepatocellular carcinoma: a randomized controlled noninferiority trial. Eur Radiol. 2023;33(6):4344–54. 36576547 10.1007/s00330-022-09298-x

[19] Park S, Yoon JH, Joo I, Yu MH, Kim JH, Park J, et al. Image quality in liver CT: low-dose deep learning vs standard-dose model-based iterative reconstructions. Eur Radiol. 2022;32(5):2865–74. 34821967 10.1007/s00330-021-08380-0

[20] Inoue A, Voss BA, Lee NJ, Takahashi H, Kozaka K, Heiken JP, et al. Diagnostic performance in Low- and High-Contrast tasks of an Image-Based denoising algorithm applied to radiation Dose-Reduced multiphase abdominal CT examinations. AJR Am J Roentgenol. 2023;220(1):73–85. 35731096 10.2214/AJR.22.27806

[21] Azarfar G, Ko SB, Adams SJ, Babyn PS. Applications of deep learning to reduce the need for iodinated contrast media for CT imaging: a systematic review. Int J Comput Assist Radiol Surg. 2023;18(10):1903–14. 36947337 10.1007/s11548-023-02862-w

[22] Mossa-Basha M, Zhu C, Pandhi T, Mendoza S, Azadbakht J, Safwat A, et al. Deep learning denoising improves CT perfusion image quality in the setting of lower contrast dosing: A feasibility study. AJNR Am J Neuroradiol. 2024;45(10):1468–74. 38844370 10.3174/ajnr.A8367 PMC11448976

[23] Wang L, Fatemi M, Alizad A. Artificial intelligence techniques in liver cancer. Front Oncol. 2024;14:1415859. 39290245 10.3389/fonc.2024.1415859 PMC11405163

[24] Tabrizian P, Holzner ML, Mehta N, Halazun K, Agopian VG, Yao F, et al. Ten-Year outcomes of liver transplant and downstaging for hepatocellular carcinoma. JAMA Surg. 2022;157(9):779–88. 35857294 10.1001/jamasurg.2022.2800 PMC9301590

[25] Faul F, Erdfelder E, Buchner A, Lang AG. Statistical power analyses using G*Power 3.1: tests for correlation and regression analyses. Behav Res Methods. 2009;41(4):1149–60. 19897823 10.3758/BRM.41.4.1149

[26] Svalkvist A, Svensson S, Hagberg T, Båth M. Viewdex 3.0—recent development of a software application facilitating assessment of image quality and observer performance. Radiat Prot Dosimetry. 2021;195(3–4):372–7. 33683321 10.1093/rpd/ncab014 PMC8507463

[27] Hoeijmakers EJI, Martens B, Hendriks BMF, Mihl C, Miclea RL, Backes WH, et al. How subjective CT image quality assessment becomes surprisingly reliable: pairwise comparisons instead of likert scale. Eur Radiol. 2024;34(7):4494–503. 38165429 10.1007/s00330-023-10493-7 PMC11213789

[28] Hong JH, Park EA, Lee W, Ahn C, Kim JH. Incremental image noise reduction in coronary CT angiography using a deep Learning-Based technique with iterative reconstruction. Korean J Radiol. 2020;21(10):1165–77. 32729262 10.3348/kjr.2020.0020 PMC7458859

[29] Nam JG, Ahn C, Choi H, Hong W, Park J, Kim JH, et al. Image quality of ultralow-dose chest CT using deep learning techniques: potential superiority of vendor-agnostic post-processing over vendor-specific techniques. Eur Radiol. 2021;31(7):5139–47. 33415436 10.1007/s00330-020-07537-7

[30] Lee S, Choi YH, Cho YJ, Lee SB, Cheon J-E, Kim WS, et al. Noise reduction approach in pediatric abdominal CT combining deep learning and dual-energy technique. Eur Radiol. 2021;31(4):2218–26. 33030573 10.1007/s00330-020-07349-9

[31] Gorenstein L, Onn A, Green M, Mayer A, Segev S, Marom EM. A novel artificial intelligence based denoising method for Ultra-Low dose CT used for lung cancer screening. Acad Radiol. 2023;30(11):2588–97. 37019699 10.1016/j.acra.2023.02.019

[32] Akagi M, Nakamura Y, Higaki T, Narita K, Honda Y, Zhou J, et al. Deep learning reconstruction improves image quality of abdominal ultra-high-resolution CT. Eur Radiol. 2019;29(11):6163–71. 30976831 10.1007/s00330-019-06170-3

[33] Nakamura Y, Higaki T, Tatsugami F, Zhou J, Yu Z, Akino N, et al. Deep Learning-based CT image reconstruction: initial evaluation targeting hypovascular hepatic metastases. Radiol Artif Intell. 2019;1(6):e180011. 33937803 10.1148/ryai.2019180011 PMC8017421

[34] Ronot M, Leporq B, van Beers BE, Vilgrain V. CT and MR perfusion techniques to assess diffuse liver disease. Abdom Radiol (New York). 2020;45(11):3496–506. 10.1007/s00261-019-02338-z 31768595

[35] Topcuoğlu OM, Karçaaltıncaba M, Akata D, Özmen MN. Reproducibility and variability of very low dose hepatic perfusion CT in metastatic liver disease. Diagn Interv Radiol (Ankara Turkey). 2016;22(6):495–500. 10.5152/dir.2016.16612 PMC5098942 27759566

[36] Lee DH, Lee JM, Baek JH, Shin CI, Han JK, Choi BI. Diagnostic performance of Gadoxetic acid-enhanced liver MR imaging in the detection of HCCs and allocation of transplant recipients on the basis of the Milan criteria and UNOS guidelines: correlation with histopathologic findings. Radiology. 2015;274(1):149–60. 25203131 10.1148/radiol.14140141

[37] Rostambeigi N, Taylor AJ, Golzarian J, Jensen EH, Pruett TL, Dudeja V, et al. Effect of MRI versus MDCT on Milan criteria scores and liver transplantation eligibility. AJR Am J Roentgenol. 2016;206(4):726–33. 26796867 10.2214/AJR.15.14642

[38] Kaufmann S, Horger T, Oelker A, Kloth C, Nikolaou K, Schulze M, et al. Characterization of hepatocellular carcinoma (HCC) lesions using a novel CT-based volume perfusion (VPCT) technique. Eur J Radiol. 2015;84(6):1029–35. 25816994 10.1016/j.ejrad.2015.02.020

[39] Kurucay M, Kloth C, Kaufmann S, Nikolaou K, Bosmuller H, Horger M, et al. Multiparametric imaging for detection and characterization of hepatocellular carcinoma using Gadoxetic acid-enhanced MRI and perfusion-CT: which parameters work best? Cancer Imaging. 2017;17(1):18. 28659180 10.1186/s40644-017-0121-9 PMC5490162

[40] Fischer MA, Kartalis N, Grigoriadis A, Loizou L, Stal P, Leidner B, et al. Perfusion computed tomography for detection of hepatocellular carcinoma in patients with liver cirrhosis. Eur Radiol. 2015;25(11):3123–32. 25903707 10.1007/s00330-015-3732-1

[41] Gordic S, Puippe GD, Krauss B, Klotz E, Desbiolles L, Lesurtel M, et al. Correlation between Dual-Energy and perfusion CT in patients with hepatocellular carcinoma. Radiology. 2016;280(1):78–87. 26824712 10.1148/radiol.2015151560

[42] Choi H, Chang W, Kim JH, Ahn C, Lee H, Kim HY, et al. Dose reduction potential of vendor-agnostic deep learning model in comparison with deep learning-based image reconstruction algorithm on CT: a Phantom study. Eur Radiol. 2022;32(2):1247–55. 34390372 10.1007/s00330-021-08199-9 PMC8364308

[43] Dehdab R, Brendel JM, Streich S, Ladurner R, Stenzl B, Mueck J, et al. Evaluation of a deep learning denoising algorithm for dose reduction in Whole-Body Photon-Counting CT imaging: A cadaveric study. Acad Radiol. 2025;32(6):3519–31. 39818525 10.1016/j.acra.2024.12.052

