# Supplementary material for: AI-based denoising improves image quality in HCC volume perfusion CT without affecting Milan classification
Source: BMC Med Imaging. 2026 Jan 2;26:53. doi: 10.1186/s12880-025-02138-6 (PMC12849736; doi:10.1186/s12880-025-02138-6)
Supplement: Supplementary file 1 — Supplementary Material 1 [file 12880_2025_2138_MOESM1_ESM.docx]

# Supplementary material:

**Table S1.** Rater-specific subjective image quality ratings and reliability/agreement metrics.

|  |  |  | | ***Intra-Rater Reliability and Inter-Rater-Agreement (Spearman's Rho), with 95% Confidence Intervals*** | | | | | |
| --- | --- | --- | --- | --- | --- | --- | --- | --- | --- |
|  |  | **Rating** | | ***Original*** | | ***Vendor*** | | ***AID*** | |
|  | **Dataset** | Rater | Mean ± SD | Rater 1 | Rater 2 | Rater 1 | Rater 2 | Rater 1 | Rater 2 |
|  |  |  |  |  |  |  |  |  |  |
| Image Quality | Original | Rater 1 | -0.34 ± 0.56 | 1.00 (0.00 to 0.00) | 0.87 (0.84 to 0.90) | 0.57 (0.49 to 0.64) | 0.52 (0.43 to 0.60) | 0.44 (0.34 to 0.53) | 0.42 (0.32 to 0.52) |
|  |  | Rater 2 | -0.28 ± 0.59 | 0.87 (0.84 to 0.90) | 1.00 (0.00 to 0.00) | 0.54 (0.46 to 0.62) | 0.49 (0.40 to 0.58) | 0.42 (0.32 to 0.51) | 0.38 (0.28 to 0.48) |
|  |  |  |  |  |  |  |  |  |  |
|  | Vendor | Rater 1 | -0.17 ± 0.59 | 0.57 (0.49 to 0.64) | 0.54 (0.46 to 0.62) | 1.00 (0.00 to 0.00) | 0.93 (0.91 to 0.94) | 0.61 (0.54 to 0.68) | 0.52 (0.43 to 0.60) |
|  |  | Rater 2 | -0.16 ± 0.60 | 0.52 (0.43 to 0.60) | 0.49 (0.40 to 0.58) | 0.93 (0.91 to 0.94) | 1.00 (0.00 to 0.00) | 0.57 (0.48 to 0.64) | 0.48 (0.39 to 0.57) |
|  |  |  |  |  |  |  |  |  |  |
|  | AID | Rater 1 | 0.52 ± 0.54 | 0.44 (0.34 to 0.53) | 0.42 (0.32 to 0.51) | 0.61 (0.54 to 0.68) | 0.57 (0.48 to 0.64) | 1.00 (0.00 to 0.00) | 0.87 (0.83 to 0.89) |
|  |  | Rater 2 | 0.45 ± 0.60 | 0.42 (0.32 to 0.52) | 0.38 (0.28 to 0.48) | 0.52 (0.43 to 0.60) | 0.48 (0.39 to 0.57) | 0.87 (0.83 to 0.89) | 1.00 (0.00 to 0.00) |
|  |  |  |  |  |  |  |  |  |  |
| Diagnostic Confidence | Original | Rater 1 | -0.18 ± 0.46 | 1.00 (0.00 to 0.00) | 0.97 (0.96 to 0.98) | 0.53 (0.44 to 0.61) | 0.47 (0.38 to 0.56) | 0.19 (0.08 to 0.30) | 0.14 (0.02 to 0.25) |
|  |  | Rater 2 | -0.19 ± 0.46 | 0.97 (0.96 to 0.98) | 1.00 (0.00 to 0.00) | 0.53 (0.44 to 0.61) | 0.47 (0.38 to 0.56) | 0.17 (0.06 to 0.29) | 0.12 (0.00 to 0.23) |
|  |  |  |  |  |  |  |  |  |  |
|  | Vendor | Rater 1 | -0.10 ± 0.43 | 0.53 (0.44 to 0.61) | 0.53 (0.44 to 0.61) | 1.00 (0.00 to 0.00) | 0.86 (0.83 to 0.89) | 0.30 (0.19 to 0.40) | 0.28 (0.16 to 0.38) |
|  |  | Rater 2 | -0.09 ± 0.44 | 0.47 (0.38 to 0.56) | 0.47 (0.38 to 0.56) | 0.86 (0.83 to 0.89) | 1.00 (0.00 to 0.00) | 0.25 (0.14 to 0.36) | 0.23 (0.12 to 0.34) |
|  |  |  |  |  |  |  |  |  |  |
|  | AID | Rater 1 | 0.29 ± 0.48 | 0.19 (0.08 to 0.30) | 0.17 (0.06 to 0.29) | 0.30 (0.19 to 0.40) | 0.25 (0.14 to 0.36) | 1.00 (0.00 to 0.00) | 0.87 (0.84 to 0.90) |
|  |  | Rater 2 | 0.25 ± 0.50 | 0.14 (0.02 to 0.25) | 0.12 (0.00 to 0.23) | 0.28 (0.16 to 0.38) | 0.23 (0.12 to 0.34) | 0.87 (0.84 to 0.90) | 1.00 (0.00 to 0.00) |
|  |  |  |  |  |  |  |  |  |  |
| Contrast | Original | Rater 1 | -0.37 ± 0.54 | 1.00 (0.00 to 0.00) | 0.93 (0.92 to 0.95) | 0.53 (0.44 to 0.61) | 0.49 (0.40 to 0.58) | 0.46 (0.37 to 0.55) | 0.36 (0.25 to 0.46) |
|  |  | Rater 2 | -0.36 ± 0.55 | 0.93 (0.92 to 0.95) | 1.00 (0.00 to 0.00) | 0.49 (0.40 to 0.57) | 0.45 (0.36 to 0.54) | 0.44 (0.34 to 0.53) | 0.34 (0.23 to 0.44) |
|  |  |  |  |  |  |  |  |  |  |
|  | Vendor | Rater 1 | -0.10 ± 0.56 | 0.53 (0.44 to 0.61) | 0.49 (0.40 to 0.57) | 1.00 (0.00 to 0.00) | 0.90 (0.87 to 0.92) | 0.50 (0.41 to 0.59) | 0.44 (0.34 to 0.53) |
|  |  | Rater 2 | -0.09 ± 0.57 | 0.49 (0.40 to 0.58) | 0.45 (0.36 to 0.54) | 0.90 (0.87 to 0.92) | 1.00 (0.00 to 0.00) | 0.47 (0.37 to 0.56) | 0.41 (0.30 to 0.50) |
|  |  |  |  |  |  |  |  |  |  |
|  | AID | Rater 1 | 0.48 ± 0.53 | 0.46 (0.37 to 0.55) | 0.44 (0.34 to 0.53) | 0.50 (0.41 to 0.59) | 0.47 (0.37 to 0.56) | 1.00 (0.00 to 0.00) | 0.84 (0.80 to 0.87) |
|  |  | Rater 2 | 0.41 ± 0.59 | 0.36 (0.25 to 0.46) | 0.34 (0.23 to 0.44) | 0.44 (0.34 to 0.53) | 0.41 (0.30 to 0.50) | 0.84 (0.80 to 0.87) | 1.00 (0.00 to 0.00) |
|  |  |  |  |  |  |  |  |  |  |
| Sharpness | Original | Rater 1 | -0.34 ± 0.53 | 1.00 (0.00 to 0.00) | 0.93 (0.91 to 0.94) | 0.59 (0.51 to 0.66) | 0.55 (0.46 to 0.63) | 0.46 (0.37 to 0.55) | 0.46 (0.37 to 0.55) |
|  |  | Rater 2 | -0.31 ± 0.54 | 0.93 (0.91 to 0.94) | 1.00 (0.00 to 0.00) | 0.55 (0.46 to 0.63) | 0.51 (0.42 to 0.59) | 0.43 (0.33 to 0.52) | 0.44 (0.34 to 0.53) |
|  |  |  |  |  |  |  |  |  |  |
|  | Vendor | Rater 1 | -0.15 ± 0.55 | 0.59 (0.51 to 0.66) | 0.55 (0.46 to 0.63) | 1.00 (0.00 to 0.00) | 0.94 (0.92 to 0.95) | 0.52 (0.42 to 0.60) | 0.48 (0.38 to 0.56) |
|  |  | Rater 2 | -0.14 ± 0.56 | 0.55 (0.46 to 0.63) | 0.51 (0.42 to 0.59) | 0.94 (0.92 to 0.95) | 1.00 (0.00 to 0.00) | 0.48 (0.39 to 0.56) | 0.44 (0.34 to 0.53) |
|  |  |  |  |  |  |  |  |  |  |
|  | AID | Rater 1 | 0.49 ± 0.53 | 0.46 (0.37 to 0.55) | 0.43 (0.33 to 0.52) | 0.52 (0.42 to 0.60) | 0.48 (0.39 to 0.56) | 1.00 (0.00 to 0.00) | 0.91 (0.89 to 0.93) |
|  |  | Rater 2 | 0.46 ± 0.55 | 0.46 (0.37 to 0.55) | 0.44 (0.34 to 0.53) | 0.48 (0.38 to 0.56) | 0.44 (0.34 to 0.53) | 0.91 (0.89 to 0.93) | 1.00 (0.00 to 0.00) |

SD = standard deviation; AID - AI Denoising

| **Table S2:** Rater-specific subjective image quality ratings and reliability/agreement metrics | | | | | | | | | | | | | | |
| --- | --- | --- | --- | --- | --- | --- | --- | --- | --- | --- | --- | --- | --- | --- |
|  |  |  |  |  |  |  |  |  |  |  |  |  |  |  |
|  |  |  |  | | |  | ***Intra-Rater Reliability and Inter-Rater-Agreement (Bayesian PABAK with 95% HDI)*** | | | | | | | |
|  |  |  | **Rating** | | |  | ***Original*** | |  | ***Vendor*** | |  | ***AID*** | |
|  | **Dataset** |  | Rater |  | Mean ± SD |  | Rater 1 | Rater 2 |  | Rater 1 | Rater 2 |  | Rater 1 | Rater 2 |
|  |  |  |  |  |  |  |  |  |  |  |  |  |  |  |
| Image Quality | Original |  | Rater 1 |  | -0.34 ± 0.56 |  | 1.00 (1.00 to 1.00) | 0.99 (0.97 to 1.00) |  | 1.00 (1.00 to 1.00) | 0.98 (0.94 to 1.00) |  | 1.00 (1.00 to 1.00) | 0.94 (0.87 to 0.99) |
|  |  |  | Rater 2 |  | -0.30 ± 0.58 |  | 0.99 (0.97 to 1.00) | 1.00 (1.00 to 1.00) |  | 0.98 (0.94 to 1.00) | 1.00 (1.00 to 1.00) |  | 0.94 (0.87 to 0.99) | 1.00 (1.00 to 1.00) |
|  |  |  |  |  |  |  |  |  |  |  |  |  |  |  |
|  | Vendor |  | Rater 1 |  | -0.18 ± 0.59 |  | 1.00 (1.00 to 1.00) | 0.98 (0.94 to 1.00) |  | 1.00 (1.00 to 1.00) | 0.99 (0.97 to 1.00) |  | 1.00 (1.00 to 1.00) | 0.89 (0.80 to 0.96) |
|  |  |  | Rater 2 |  | -0.16 ± 0.60 |  | 0.98 (0.94 to 1.00) | 1.00 (1.00 to 1.00) |  | 0.99 (0.97 to 1.00) | 1.00 (1.00 to 1.00) |  | 0.89 (0.80 to 0.96) | 1.00 (1.00 to 1.00) |
|  |  |  |  |  |  |  |  |  |  |  |  |  |  |  |
|  | AID |  | Rater 1 |  | 0.53 ± 0.54 |  | 1.00 (1.00 to 1.00) | 0.92 (0.85 to 0.98) |  | 1.00 (1.00 to 1.00) | 0.94 (0.88 to 0.99) |  | 1.00 (1.00 to 1.00) | 0.99 (0.97 to 1.00) |
|  |  |  | Rater 2 |  | 0.48 ± 0.58 |  | 0.92 (0.85 to 0.98) | 1.00 (1.00 to 1.00) |  | 0.94 (0.88 to 0.99) | 1.00 (1.00 to 1.00) |  | 0.99 (0.97 to 1.00) | 1.00 (1.00 to 1.00) |
|  |  |  |  |  |  |  |  |  |  |  |  |  |  |  |
| Diagnostic Confidence | Original |  | Rater 1 |  | -0.08 ± 0.37 |  | 1.00 (1.00 to 1.00) | 0.99 (0.97 to 1.00) |  | 1.00 (1.00 to 1.00) | 0.98 (0.94 to 1.00) |  | 1.00 (1.00 to 1.00) | 0.94 (0.88 to 0.99) |
|  |  |  | Rater 2 |  | -0.07 ± 0.37 |  | 0.99 (0.97 to 1.00) | 1.00 (1.00 to 1.00) |  | 0.98 (0.94 to 1.00) | 1.00 (1.00 to 1.00) |  | 0.94 (0.88 to 0.99) | 1.00 (1.00 to 1.00) |
|  |  |  |  |  |  |  |  |  |  |  |  |  |  |  |
|  | Vendor |  | Rater 1 |  | -0.07 ± 0.29 |  | 1.00 (1.00 to 1.00) | 0.99 (0.97 to 1.00) |  | 1.00 (1.00 to 1.00) | 0.99 (0.97 to 1.00) |  | 1.00 (1.00 to 1.00) | 0.98 (0.93 to 1.00) |
|  |  |  | Rater 2 |  | -0.05 ± 0.29 |  | 0.99 (0.97 to 1.00) | 1.00 (1.00 to 1.00) |  | 0.99 (0.97 to 1.00) | 1.00 (1.00 to 1.00) |  | 0.98 (0.93 to 1.00) | 1.00 (1.00 to 1.00) |
|  |  |  |  |  |  |  |  |  |  |  |  |  |  |  |
|  | AID |  | Rater 1 |  | 0.15 ± 0.38 |  | 1.00 (1.00 to 1.00) | 0.96 (0.90 to 1.00) |  | 1.00 (1.00 to 1.00) | 0.98 (0.94 to 1.00) |  | 1.00 (1.00 to 1.00) | 0.99 (0.97 to 1.00) |
|  |  |  | Rater 2 |  | 0.13 ± 0.39 |  | 0.96 (0.90 to 1.00) | 1.00 (1.00 to 1.00) |  | 0.98 (0.94 to 1.00) | 1.00 (1.00 to 1.00) |  | 0.99 (0.97 to 1.00) | 1.00 (1.00 to 1.00) |
|  |  |  |  |  |  |  |  |  |  |  |  |  |  |  |
| Contrast | Original |  | Rater 1 |  | -0.35 ± 0.54 |  | 1.00 (1.00 to 1.00) | 0.99 (0.97 to 1.00) |  | 1.00 (1.00 to 1.00) | 0.94 (0.88 to 0.99) |  | 1.00 (1.00 to 1.00) | 0.94 (0.87 to 0.99) |
|  |  |  | Rater 2 |  | -0.33 ± 0.55 |  | 0.99 (0.97 to 1.00) | 1.00 (1.00 to 1.00) |  | 0.94 (0.88 to 0.99) | 1.00 (1.00 to 1.00) |  | 0.94 (0.87 to 0.99) | 1.00 (1.00 to 1.00) |
|  |  |  |  |  |  |  |  |  |  |  |  |  |  |  |
|  | Vendor |  | Rater 1 |  | -0.13 ± 0.56 |  | 1.00 (1.00 to 1.00) | 0.94 (0.88 to 0.99) |  | 1.00 (1.00 to 1.00) | 0.99 (0.97 to 1.00) |  | 1.00 (1.00 to 1.00) | 0.92 (0.85 to 0.98) |
|  |  |  | Rater 2 |  | -0.15 ± 0.56 |  | 0.94 (0.88 to 0.99) | 1.00 (1.00 to 1.00) |  | 0.99 (0.97 to 1.00) | 1.00 (1.00 to 1.00) |  | 0.92 (0.85 to 0.98) | 1.00 (1.00 to 1.00) |
|  |  |  |  |  |  |  |  |  |  |  |  |  |  |  |
|  | AID |  | Rater 1 |  | 0.50 ± 0.53 |  | 1.00 (1.00 to 1.00) | 0.96 (0.90 to 1.00) |  | 1.00 (1.00 to 1.00) | 0.99 (0.97 to 1.00) |  | 1.00 (1.00 to 1.00) | 0.99 (0.97 to 1.00) |
|  |  |  | Rater 2 |  | 0.45 ± 0.57 |  | 0.96 (0.90 to 1.00) | 1.00 (1.00 to 1.00) |  | 0.99 (0.97 to 1.00) | 1.00 (1.00 to 1.00) |  | 0.99 (0.97 to 1.00) | 1.00 (1.00 to 1.00) |
|  |  |  |  |  |  |  |  |  |  |  |  |  |  |  |
| Sharpness | Original |  | Rater 1 |  | -0.35 ± 0.53 |  | 1.00 (1.00 to 1.00) | 0.99 (0.97 to 1.00) |  | 1.00 (1.00 to 1.00) | 0.94 (0.87 to 0.99) |  | 1.00 (1.00 to 1.00) | 0.90 (0.82 to 0.97) |
|  |  |  | Rater 2 |  | -0.35 ± 0.53 |  | 0.99 (0.97 to 1.00) | 1.00 (1.00 to 1.00) |  | 0.94 (0.87 to 0.99) | 1.00 (1.00 to 1.00) |  | 0.90 (0.82 to 0.97) | 1.00 (1.00 to 1.00) |
|  |  |  |  |  |  |  |  |  |  |  |  |  |  |  |
|  | Vendor |  | Rater 1 |  | -0.15 ± 0.57 |  | 1.00 (1.00 to 1.00) | 0.94 (0.87 to 0.99) |  | 1.00 (1.00 to 1.00) | 0.99 (0.97 to 1.00) |  | 1.00 (1.00 to 1.00) | 0.92 (0.84 to 0.98) |
|  |  |  | Rater 2 |  | -0.13 ± 0.57 |  | 0.94 (0.87 to 0.99) | 1.00 (1.00 to 1.00) |  | 0.99 (0.97 to 1.00) | 1.00 (1.00 to 1.00) |  | 0.92 (0.84 to 0.98) | 1.00 (1.00 to 1.00) |
|  |  |  |  |  |  |  |  |  |  |  |  |  |  |  |
|  | AID |  | Rater 1 |  | 0.50 ± 0.53 |  | 1.00 (1.00 to 1.00) | 0.94 (0.88 to 0.99) |  | 1.00 (1.00 to 1.00) | 0.94 (0.87 to 0.99) |  | 1.00 (1.00 to 1.00) | 0.99 (0.97 to 1.00) |
|  |  |  | Rater 2 |  | 0.44 ± 0.58 |  | 0.94 (0.88 to 0.99) | 1.00 (1.00 to 1.00) |  | 0.94 (0.87 to 0.99) | 1.00 (1.00 to 1.00) |  | 0.99 (0.97 to 1.00) | 1.00 (1.00 to 1.00) |
|  |  |  |  |  |  |  |  |  |  |  |  |  |  |  |
| PABAK = Prevalence‐adjusted Bias‐adjusted Kappa; HDI = Highest Density Interval; SD = standard deviation; AID - AI Denoising | | | | | | | | | | | | | | |

**Table S3.** Pooled subjective image quality ratings and corrected pairwise comparisons

|  | Dataset |  | Rating (pooled) |  | Significance (p) | | | | |
| --- | --- | --- | --- | --- | --- | --- | --- | --- | --- |
|  |  |  | (Mean ± SD) |  | Original |  | Vendor |  | AID |
|  |  |  |  |  |  |  |  |  |  |
| Image Quality | Original |  | -0.31 ± 0.29 |  | n/a |  | 0.023 |  | <0.001 |
|  |  |  |  |  |  |  |  |  |  |
|  | Vendor |  | -0.17 ± 0.24 |  | 0.023 |  | n/a |  | <0.001 |
|  |  |  |  |  |  |  |  |  |  |
|  | AID |  | 0.48 ± 0.29 |  | <0.001 |  | <0.001 |  | n/a |
|  |  |  |  |  |  |  |  |  |  |
| Diagnostic Confidence | Original |  | -0.19 ± 0.26 |  | n/a |  | 0.153 |  | <0.001 |
|  |  |  |  |  |  |  |  |  |  |
|  | Vendor |  | -0.10 ± 0.22 |  | 0.153 |  | n/a |  | <0.001 |
|  |  |  |  |  |  |  |  |  |  |
|  | AID |  | 0.27 ± 0.31 |  | <0.001 |  | <0.001 |  | n/a |
|  |  |  |  |  |  |  |  |  |  |
| Contrast | Original |  | -0.36 ± 0.26 |  | n/a |  | <0.001 |  | <0.001 |
|  |  |  |  |  |  |  |  |  |  |
|  | Vendor |  | -0.10 ± 0.24 |  | <0.001 |  | n/a |  | <0.001 |
|  |  |  |  |  |  |  |  |  |  |
|  | AID |  | 0.44 ± 0.26 |  | <0.001 |  | <0.001 |  | n/a |
|  |  |  |  |  |  |  |  |  |  |
| Sharpness | Original |  | -0.33 ± 0.26 |  | n/a |  | <0.001 |  | <0.001 |
|  |  |  |  |  |  |  |  |  |  |
|  | Vendor |  | -0.14 ± 0.23 |  | <0.001 |  | n/a |  | <0.001 |
|  |  |  |  |  |  |  |  |  |  |
|  | AID |  | 0.47 ± 0.27 |  | <0.001 |  | <0.001 |  | n/a |

SD = standard deviation; AID = AI Denoising; n/a = not applicable

**Table S4.** Objective image quality metrics and corrected pairwise comparisons

|  | **Dataset** |  | **Rating (pooled)** |  | ***Significance (p)*** | | | | |
| --- | --- | --- | --- | --- | --- | --- | --- | --- | --- |
|  |  |  | (Mean ± SD) |  | Original |  | Vendor |  | AID |
|  |  |  |  |  |  |  |  |  |  |
| HU Aorta | Original |  | 619.19 ± 6.95 |  | n/a |  | >0.999 |  | 0.253 |
|  |  |  |  |  |  |  |  |  |  |
|  | Vendor |  | 619.23 ± 6.76 |  | >0.999 |  | n/a |  | 0.608 |
|  |  |  |  |  |  |  |  |  |  |
|  | AID |  | 619.39 ± 6.50 |  | 0.253 |  | 0.608 |  | n/a |
|  |  |  |  |  |  |  |  |  |  |
| HU Muscle | Original |  | 46.13 ±3.60 |  | n/a |  | 0.914 |  | 0.122 |
|  |  |  |  |  |  |  |  |  |  |
|  | Vendor |  | 46.26 ± 3.46 |  | 0.914 |  | n/a |  | 0.992 |
|  |  |  |  |  |  |  |  |  |  |
|  | AID |  | 46.36 ± 3.33 |  | 0.122 |  | 0.992 |  | n/a |
|  |  |  |  |  |  |  |  |  |  |
| SD Aorta | Original |  | 40.44 ± 3.83 |  | n/a |  | <0.001 |  | <0.001 |
|  |  |  |  |  |  |  |  |  |  |
|  | Vendor |  | 35.29 ± 3.51 |  | <0.001 |  | n/a |  | <0.001 |
|  |  |  |  |  |  |  |  |  |  |
|  | AID |  | 25.01 ± 2.95 |  | <0.001 |  | <0.001 |  | n/a |
|  |  |  |  |  |  |  |  |  |  |
| SD Muscle | Original |  | 27.65 ± 2.74 |  | n/a |  | <0.001 |  | <0.001 |
|  |  |  |  |  |  |  |  |  |  |
|  | Vendor |  | 24.22 ± 2.31 |  | <0.001 |  | n/a |  | <0.001 |
|  |  |  |  |  |  |  |  |  |  |
|  | AID |  | 17.38 ± 2.27 |  | <0.001 |  | <0.001 |  | n/a |
|  |  |  |  |  |  |  |  |  |  |
| CNR | Original |  | 16.98 ± 1.54 |  | n/a |  | <0.001 |  | <0.001 |
|  |  |  |  |  |  |  |  |  |  |
|  | Vendor |  | 19.43 ± 1.79 |  | <0.001 |  | n/a |  | <0.001 |
|  |  |  |  |  |  |  |  |  |  |
|  | AID |  | 27.40 ± 2.98 |  | <0.001 |  | <0.001 |  | n/a |

HU = Hounsfiled Unit; SD = standard deviation; CNR = Contrast-to-noise ratio; AID = AI Denoising; n/a = not applicable

**Table S5.** Lesion diameter and corrected pairwise comparisons.

| Liver Segments | Dataset |  | Lesions |  | Lesion Diameter |  | p (Significance) | | | | |
| --- | --- | --- | --- | --- | --- | --- | --- | --- | --- | --- | --- |
|  |  |  | n |  | Mean ± SD (mm) |  | Original |  | Vendor |  | AID |
|  |  |  |  |  |  |  |  |  |  |  |  |
| I | Original |  | 3 |  | 17 ± 4 |  | n/a |  | >0.999 |  | >0.999 |
|  | Vendor |  | 3 |  | 16 ± 4 |  | >0.999 |  | n/a |  | >0.999 |
|  | AID |  | 3 |  | 16 ± 4 |  | >0.999 |  | >0.999 |  | n/a |
|  |  |  |  |  |  |  |  |  |  |  |  |
| II | Original |  | 9 |  | 32 ± 14 |  | n/a |  | >0.999 |  | >0.999 |
|  | Vendor |  | 9 |  | 32 ± 15 |  | >0.999 |  | n/a |  | >0.999 |
|  | AID |  | 9 |  | 32 ± 15 |  | >0.999 |  | >0.999 |  | n/a |
|  |  |  |  |  |  |  |  |  |  |  |  |
| III | Original |  | 8 |  | 31 ± 15 |  | n/a |  | >0.999 |  | >0.999 |
|  | Vendor |  | 8 |  | 32 ± 16 |  | >0.999 |  | n/a |  | >0.999 |
|  | AID |  | 8 |  | 32 ± 16 |  | >0.999 |  | >0.999 |  | n/a |
|  |  |  |  |  |  |  |  |  |  |  |  |
| Iva | Original |  | 6 |  | 37 ± 29 |  | n/a |  | >0.999 |  | >0.999 |
|  | Vendor |  | 6 |  | 40 ± 31 |  | >0.999 |  | n/a |  | >0.999 |
|  | AID |  | 6 |  | 37 ± 30 |  | >0.999 |  | >0.999 |  | n/a |
|  |  |  |  |  |  |  |  |  |  |  |  |
| IVb | Original |  | 8 |  | 31 ± 15 |  | n/a |  | >0.999 |  | >0.999 |
|  | Vendor |  | 8 |  | 32 ± 16 |  | >0.999 |  | n/a |  | >0.999 |
|  | AID |  | 8 |  | 30 ± 14 |  | >0.999 |  | >0.999 |  | n/a |
|  |  |  |  |  |  |  |  |  |  |  |  |
| V | Original |  | 11 |  | 38 ± 22 |  | n/a |  | >0.999 |  | >0.999 |
|  | Vendor |  | 11 |  | 38 ± 22 |  | >0.999 |  | n/a |  | >0.999 |
|  | AID |  | 11 |  | 38 ± 21 |  | >0.999 |  | >0.999 |  | n/a |
|  |  |  |  |  |  |  |  |  |  |  |  |
| VI | Original |  | 15 |  | 35 ± 33 |  | n/a |  | >0.999 |  | >0.999 |
|  | Vendor |  | 15 |  | 35 ± 33 |  | >0.999 |  | n/a |  | >0.999 |
|  | AID |  | 15 |  | 35 ± 33 |  | >0.999 |  | >0.999 |  | n/a |
|  |  |  |  |  |  |  |  |  |  |  |  |
| VII | Original |  | 29 |  | 33 ± 33 |  | n/a |  | >0.999 |  | >0.999 |
|  | Vendor |  | 29 |  | 33 ± 33 |  | >0.999 |  | n/a |  | >0.999 |
|  | AID |  | 29 |  | 32 ± 32 |  | >0.999 |  | >0.999 |  | n/a |
|  |  |  |  |  |  |  |  |  |  |  |  |
| VIII | Original |  | 27 |  | 31 ± 27 |  | n/a |  | >0.999 |  | >0.999 |
|  | Vendor |  | 27 |  | 30 ± 27 |  | >0.999 |  | n/a |  | >0.999 |
|  | AID |  | 27 |  | 30 ± 27 |  | >0.999 |  | >0.999 |  | n/a |
